# Endoscope-Assisted Retrosigmoid Approach for Vestibular Schwannomas With Intracanalicular Extensions: Facial Nerve Outcomes

**DOI:** 10.3389/fonc.2021.774462

**Published:** 2022-01-18

**Authors:** Yunke Bi, Yunjia Ni, Dandan Gao, Qingwei Zhu, Qiangyi Zhou, Junjia Tang, Juan Liu, Fei Shi, Hongchan Li, Jian Yin, Yaohua Liu, Meiqing Lou

**Affiliations:** ^1^ Department of Neurosurgery, Shanghai General Hospital, Shanghai Jiao Tong University School of Medicine, Shanghai, China; ^2^ Department of Neurosurgery, Shanghai General Hospital of Nanjing Medical University, Shanghai, China; ^3^ Department of Hematology-oncology, Zhoupu Hospital, Shanghai University of Medicine and Health Sciences, Shanghai, China

**Keywords:** vestibular schwannomas, neuroendoscope, retrosigmoid approach, internal acoustic canal, facial nerve function

## Abstract

**Objective:**

To explore the role of neuroendoscope assistance during surgical resection of the intracanalicular portion of vestibular schwannomas *via* the retrosigmoid approach and the subsequent early facial nerve outcomes.

**Methods:**

Patients of vestibular schwannoma with intracanalicular extensions undergoing retrosigmoid dissection at a single institution were retrospectively analyzed in this study. Several surgical techniques were applied to ensure maximal and safe removal of tumors. Tumors extending less than 10 mm into the internal acoustic canal (IAC) were classified as Grade A, while those extending over 10 mm into IAC were taken as Grade B. Neuroendoscope was applied at the end of microscopic phase to search for potential remnants for Grade B tumors. Absolute tumor extension was defined and measured. House and Brackmann (HB) scale was used to evaluate immediate CN VII outcomes.

**Results:**

Of the 61 patients, there were 38 females and 23 males. A total of 18 (29.51%) cases were Koos Grade II, 12 (19.67%) cases Koos Grade III, and 31 (50.82%) cases Koos Grade IV. There were 38 cases (62.30%) of Grade A and 23 cases (37.70%) of Grade B. Gross total resection was achieved in 60 cases (98.36%). Four cases of intracanalicular remnants were detected and completely removed under endoscopic visualizations. There was a significantly higher proportion (17%, p = 0.02) of intracanalicular remnants in Grade B than Grade A. CN VII and VIII were anatomically preserved in all cases. A total of 55 cases (90.16%) retained good (HB Grades 1 and 2) facial nerve outcomes.

**Conclusions:**

In Grade B vestibular schwannomas, after maximal microsurgical removal, endoscopic evaluation of the intracanalicular portion revealed residual tumors in 17% of the patients. Hence endoscopic evaluation of the potential intracanalicular remnants for tumor extending over 10 mm within IAC (Grade B) is recommended.

## Introduction

Vestibular schwannoma is the most common benign tumor in the cerebellopontine angle (CPA). Management goal is to achieve optimal resection while preserving local structures, especially for CN VII (1–3). The retrosigmoid approach is the workhorse for management of CPA lesions. Most vestibular schwannomas present with intracanalicular extensions (4). In retrosigmoid approach, drilling the posterior wall of IAC is one of the key steps to dissect the intracanalicular portion of the tumor. However, for tumors located at the lateral end of IAC, “blind spots” under the microscope may impede gross total resection. With advances of techniques and technologies, the application of an endoscope can potentially solve the problem. However, the role of an endoscope during microsurgery has not been fully investigated. Here we report a retrospective single-operator series of 61 cases of vestibular schwannoma with intracanalicular extensions, where an endoscope was applied at the end of the microscopic phase to confirm the presence of remnants if tumor extends over 10 mm into IAC. Early facial nerve outcomes were satisfactory, and the post-operative course was uneventful.

## Materials and Methods

### Sample Selection

After approval of the institutional review board, patients with pathologically confirmed vestibular schwannoma from November 1, 2019 to December 31, 2020 at the Department of Neurosurgery, Shanghai General Hospital, Shanghai Jiao Tong University were retrospectively reviewed. Medical records, imaging studies, and surgical footages were evaluated by senior authors (JY and YL) independently; any discrepancy was discussed and solved.

### Defining the Absolute and Relative Tumor Extension Into IAC

**Figure 1:**
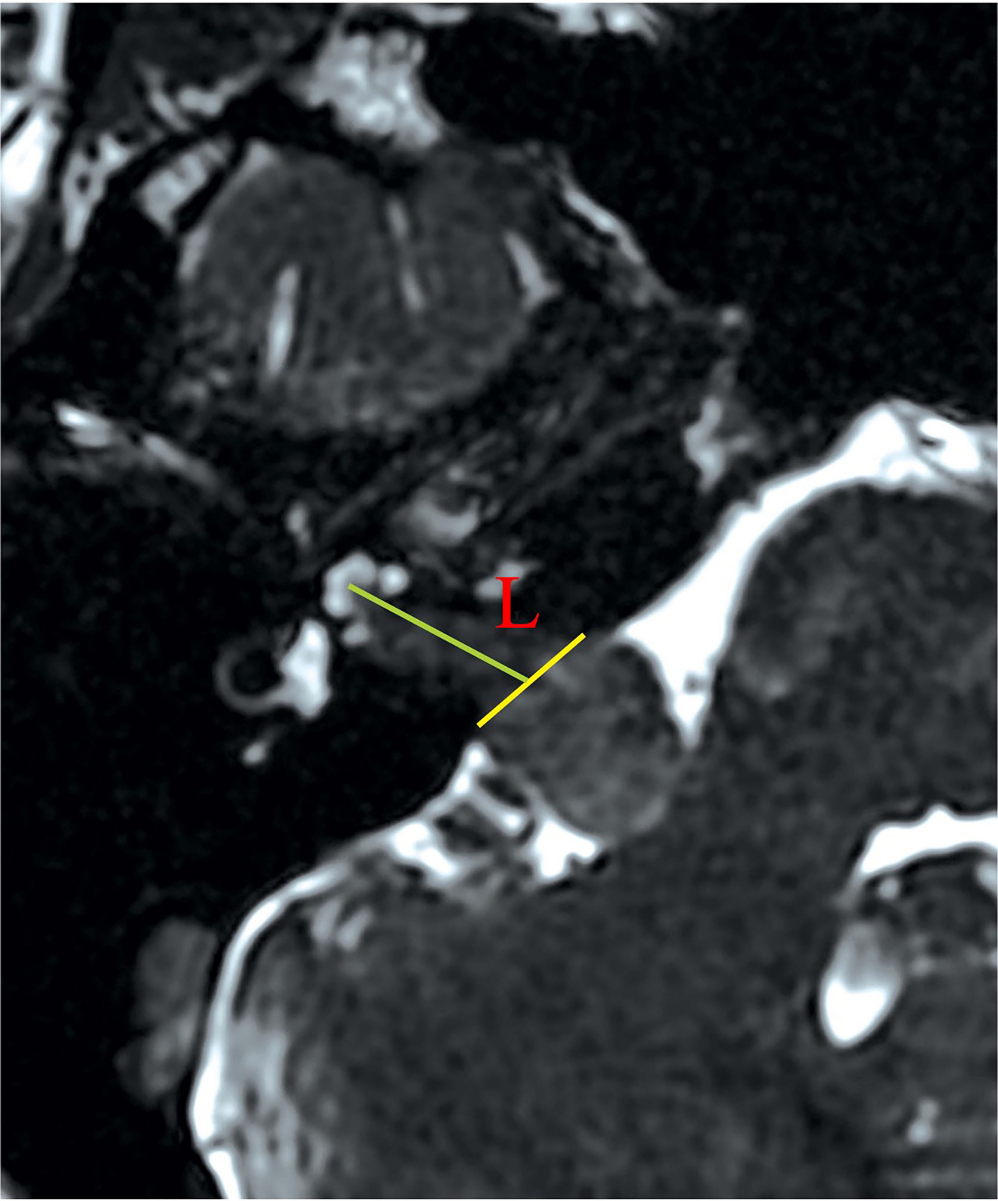
Measurement of the absolute tumor extension into IAC. On FIESTA-C, the slice with the most lateral extension of tumor is selected for measurement. An auxiliary line (yellow) is drawn between the anterior and posterior lip of the porus acusticus. The length of absolute tumor extension (labeled as L) is defined as the distance between the midpoint of the yellow line and the most lateral point of tumor extension. Defining the absolute extension of tumor in this way is different from assuming the extension of tumor as its projection on the posterior wall of IAC. The latter method appears ideal to pre-operatively define the length of the drilled posterior wall of IAC. However, the presence of tumor may erode the bony structure of the posterior wall of IAC, making delineation of the projection line difficult on pre-operative imaging studies. FIESTA-C, heavily T2-weighted fast imaging employing steady-state acquisition with cycle phase.

Imaging studies using heavily T2-weighted fast imaging employing steady-state acquisition with cycle phase (FIESTA-C) MRI were reviewed by two authors independently. A straight line (labeled as L; **Figure 1**) was drawn medially from the most lateral point of the intracanalicular portion to the midpoint between the anterior and posterior lip of the ipsilateral porus acusticus on the same axial slice. The absolute extension of the tumor into IAC was graded by the length of L, where Grade A was defined as L <10 mm and Grade B was for L ≥10 mm.

### Tumor Resection

This is a single-surgeon retrospective series in which all cases were performed by the senior author (ML) and the assistants. Since most of the procedural details have been well established in literature, they will not be exhaustive here. All patients were placed in the park bench position for retrosigmoid approach regardless of the tumor size. Neuromonitoring with a stimulus threshold of 0.1 mA was applied throughout the procedure to preserve the functions of CN VII. We started with initial debulking of the extracanalicular portion at its center and proceeded to early drill-out of the posterior wall of IAC (detailed below). After removal of the intracanalicular portion, we proceeded to decrease tumor burdens at the CPA cistern. In this paper, we focus on surgical nuances that are critical to ensure maximal tumor removal and preservation of CN VII functions.

After defining the cleavage plane, care should be taken to protect the perineurium as much as possible while performing subperineurial dissection. In practice, when the cleavage plane was initially “toward” the observer, microscopic forceps were used to peel off the perineurium. Conversely, when the cleavage plane was initially “away” from the observer, microdissectors should be used to “push away” the perineurium. In certain conditions, strict adhesions of the perineurium were sometimes encountered when neither “peeling” nor “pushing” was successful. It was then necessary to use sharp dissection by blunt-tipped microscissors. Strict adhesions were usually encountered at the porus acusticus, where CN VII was prone to inadvertent injuries. We would deal with this site at the end stage of the tumor removal procedure.

**Figure 2:**
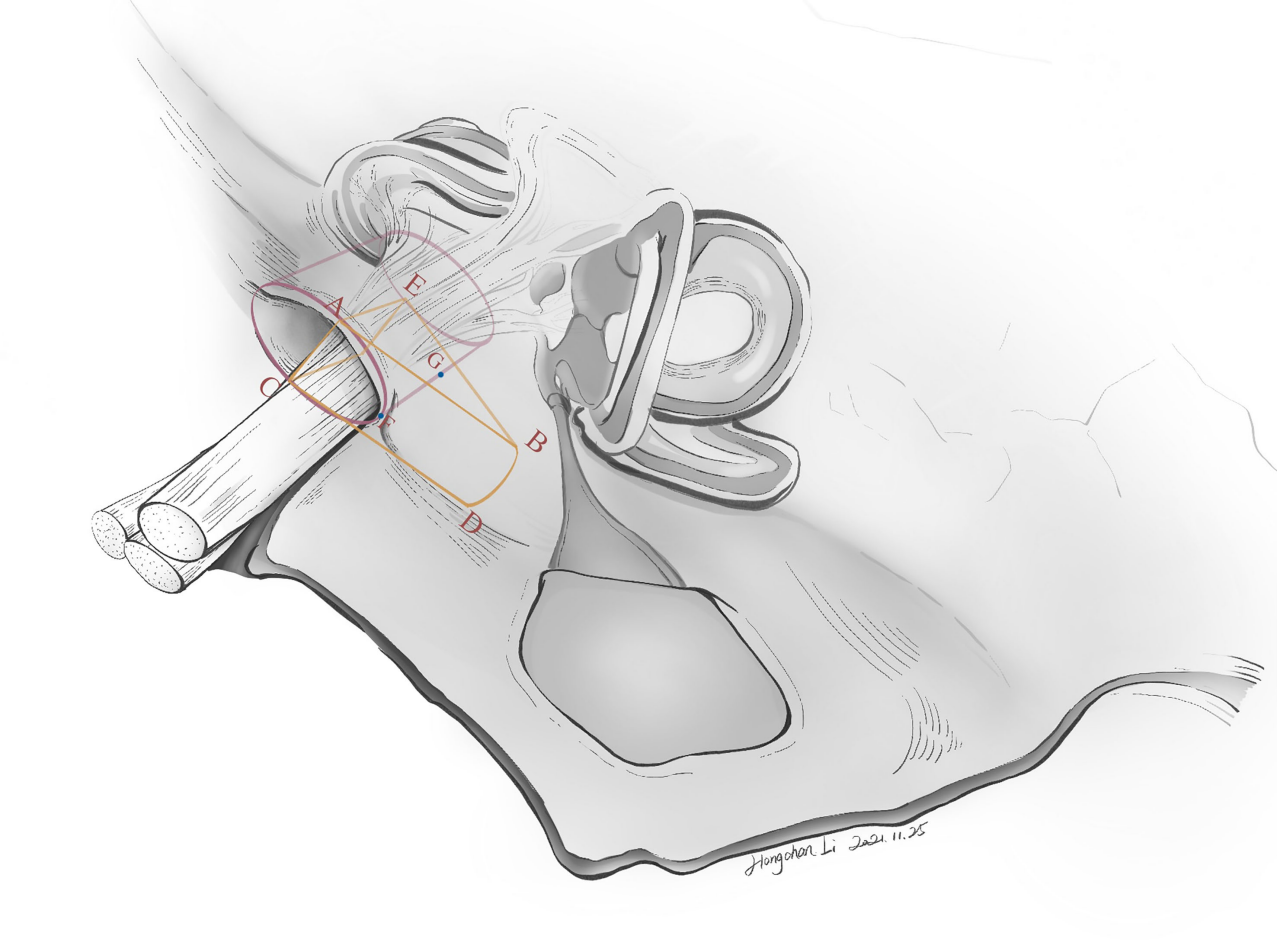
The ideal drilling can maximize tumor exposure while avoiding injuries to neighboring structures (i.e., the bony labyrinth, the endolymphatic sac). To date, neither a standardized surgical planning nor a precise drilling method has been introduced to clinical practice. However, drilling the posterior wall of IAC is not an improvised technique but has flexible rules to follow. The artist’s (Hongchan Li) illustration here shows several essential parameters in defining the safe drilling range of the posterior wall of IAC. Lines AB and CD correspond to the “entry borders” of lateral drilling, which is approximately 12–14 mm in length. This means that approximately 12–14 mm of dura along the petrosal surface is incised, after identification of the Tübingen line. ∠ABE is the drilling angle, usually 43˚. Line GF denotes the length of the drilled posterior wall of IAC, which is about 8 mm in length. Line AE denotes the antero-superior border of the drilling range. Of note, these parameters are individualized to each patient.

The drilling range depended largely on the local anatomy and intracanalicular extension of the tumor. The Tübingen line was first identified to locate the course of the posterior wall of IAC. For all Grade A and B tumors, an average of 8 mm of the posterior wall of IAC was first removed, with a drilling angle of approximately 43° (**Figure 2**). About 12–14 mm of dura was removed and incised with a No. 15 blade along the posterior petrous surface (**Figure 2**). These were not strict doctrines to follow but tangible rules to be individually adjusted. If a high-riding jugular bulb (HJB) was identified as drilling came close to it, an artificial dural substitute was used to slightly displace the HJB. We initially chose a 4 mm diamond drill bit (Medtronic plc, Minnesota, United States), and switched to smaller calibers (e.g., 2.3 mm) with further drilling. A 180˚ exposure of the posterior circumference of the porus acusticus was made. During drilling, tactile feedback on the instrument was important to indicate the proximity of semicircular canal (SCC) and the posterior wall of IAC.

**Figure 3:**
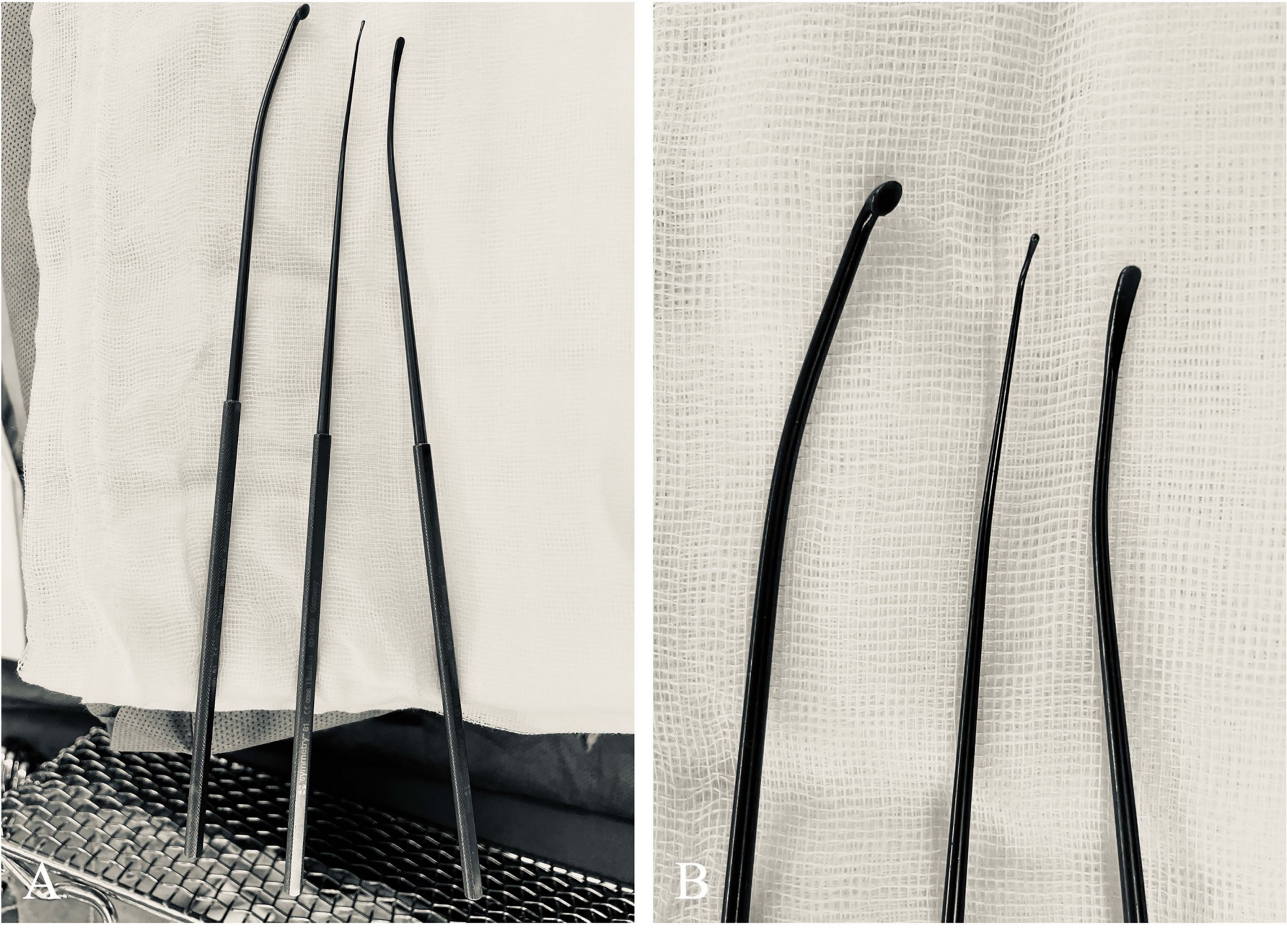
Curated angled instruments used for managing tumors located at the lateral end of IAC. **(A)** These instruments, modified from market-sold models (Symmetry Surgical GmbH, Tuttlingen, Germany), were (from left to right) microcurette, angled nerve hook, and micro-Penfield dissector. **(B)** A close-up view of the tips of these angled instruments.

Techniques of managing the intracanalicular portion were different from those of the CPA cistern. Bipolar forceps were not allowed. Following drilling of the posterior wall of IAC, dura was incised open, and we started with tumor debulking to lessen the tumor burden on nerves. After confirmation by stimulation, the vestibular nerve was sacrificed. Tumor removal was performed at the CN VIII (caudal) side, followed by the CN VII (cephalad) side. We prefer fine-tipped forceps, blunt-tipped scissors, fine-tipped suction tubes (1.2–1.5 mm) and round or flat-tipped dissectors to perform these manipulations to decrease the risk of nerve injuries. During the procedure, tumor resection was proceeded under microscopic view with pulsatile (set interval: about 2 s) irrigation of lukewarm (37°C) natural saline. Manipulating force was applied to the tumor only, and direct contact of instruments with the nerve should always be avoided. After the microscopic phase, a 0-degree neuroendoscope (2.9 mm; Karl Storz Endoskope, SE & Co. KG, Tuttlingen, Germany) would be applied to search for potential remnants in Grade B tumors. If confirmed, angled nerve hooks should usually suffice to palpate and “undress” tumor remnants. In some cases, an extra 2 mm of the posterior wall of IAC (hence a total of 10 mm) would be drilled under endoscopic view to provide space for more laterally located tumor remnants. Angled instruments, including angled nerve hooks, angled dissectors (**Figure 3**), and angled suction tubes (1.5 mm), were superior to other instruments at the endoscopic phase. These angled, personally curated instruments were modified from market-sold models (Symmetry Surgical GmbH, Tuttlingen, Germany), and were highly efficient in removing the remnants located at the fundus of IAC. To remove the tumor, we performed counter-clockwise maneuvers to separate the left-sided tumors and clockwise maneuvers for the right-sided ones. The tumor was separated from CN VII in a circular motion, with force mainly applied to the tumor body instead of CN VII.

### Evaluation of Facial Nerve Functions

Immediate post-operative facial nerve functions were graded on the House and Brackmann (HB) scale after patient awakening from anesthesia in the recovery unit.

### Statistical Analysis

Stata/SE 17.0 (StataCorp LLC, Texas, USA) was utilized for statistical analysis. Fisher’s exact test was used to calculate differences of frequencies in categorical variables. A p-value <0.05 was deemed significant to reject null hypothesis.

## Results

### Summary of the Sample

Included in this study were 61 patients (38 females and 23 males, **Table 1**). Mean (SD) age was 47.0 (14.6) years. A total of 31 (50.82%) cases were left-sided vestibular schwannomas, 30 (49.18%) right-sided. There were 18 (29.51%) cases of Koos Grade II, 12 (19.67%) cases of Koos Grade III, and 31 (50.82%) cases of Koos Grade IV. All patients were HB Grade 1 pre-operatively.

**Table 1:** Basic information of patients involved in this study.

Items	Quantity and percentage (%)			
Gender	Female	Male		
	38 (74.51)	23 (45.10)		
Tumor laterality	Left sided	Right sided		
	31 (50.82)	29 (49.18)		
Koos grade	II	III	IV	
	18 (29.51)	12(19.67)	31 (50.82)	
Resection outcome	Gross total resection	Subtotal resection		
	60 (98.36)	1 (1.64)		
IAC extension grading	A	B		
	38 (62.30)	23 (37.70)		
Post-op HB	1	2	3	4
	50 (81.97)	5 (8.20)	4 (6.56)	2 (3.28)

### Absolute Tumor Extension Into IAC

All tumors present with intracanalicular portions. Regarding the absolute tumor extension into IAC, 38 cases (62.30%) were classified as Grade A, and 23 cases (37.70%) were Grade B.

### Surgical Outcomes and Post-Operative HB Scale

**Figure 4:**
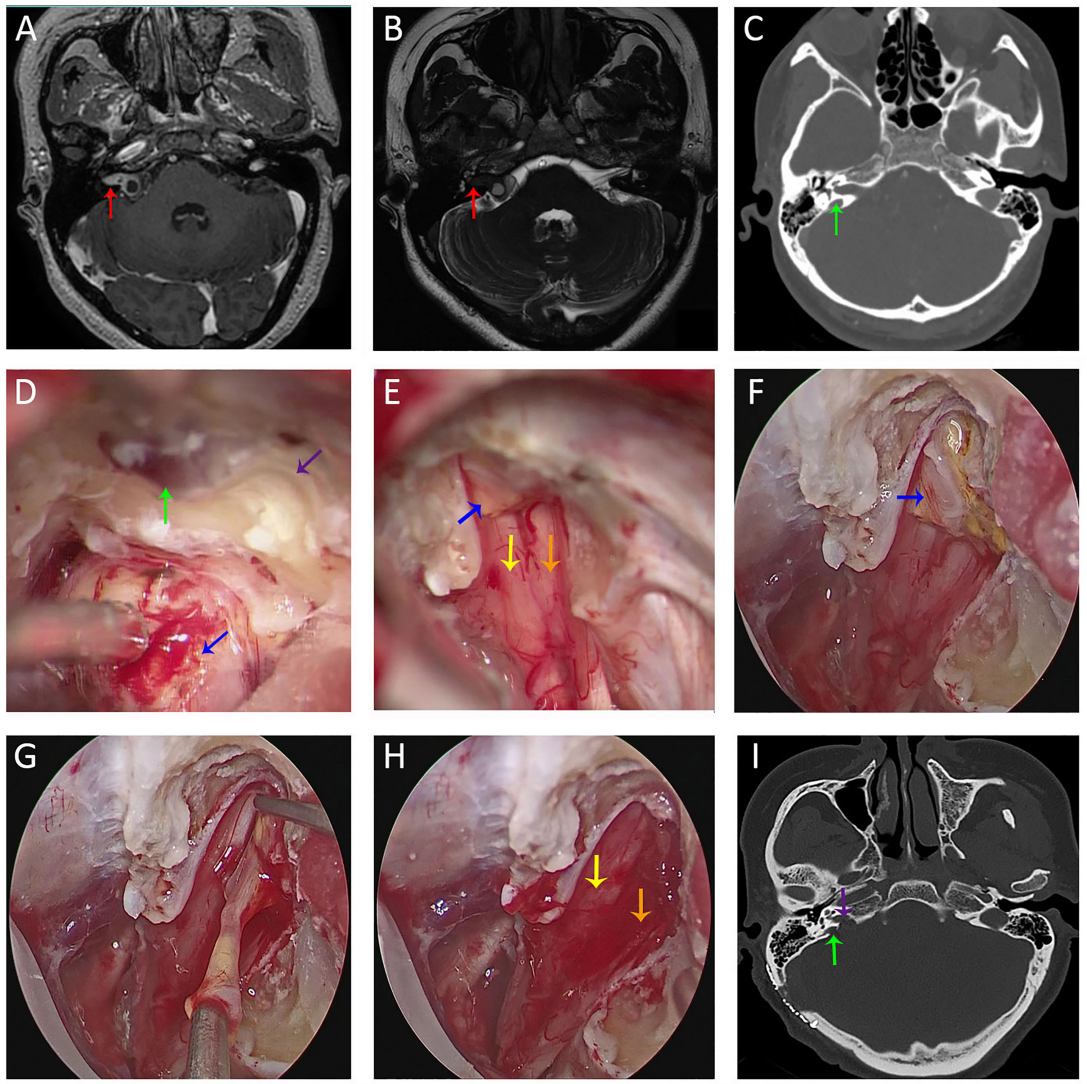
Imaging studies and intra-operative snapshots of a vestibular schwannoma patient with tumor extending into the IAC and a high-riding jugular bulb. **(A, B)** Pre-operative T1WI with contrast **(A)** and FIESTA-C **(B)** indicated a right-sided vestibular schwannoma extending into the IAC [red arrow in **(A)**]. The fundus of IAC was occupied by the tumor without CSF signal as indicated by the red arrow in **(B)**. **(C)** Pre-operative CT imaging revealed a right-sided high-riding jugular bulb (green arrow); **(D)** After debulking of the tumor at the porus acusticus (blue arrow), the posterior IAC was drilled (purple arrow) and skeletonization of the bone matter adjacent to the jugular bulb was performed. **(E)** After transposition of the jugular bulb, the posterior IAC was further drilled. After tumor removal under microscopic view, there were still tumor remnants (blue arrow) at the fundus of IAC. CN VII (yellow arrow) and CN VIII (orange arrow) were anatomically preserved. **(F)** With neuroendoscope, tumor remnants (blue arrow) were better visualized and delineated. **(G)** Tumor resection between two arachnoid layers under direct endoscopic view. **(H)** CN VII (yellow arrow) and CN VIII (orange arrow) were anatomically preserved after total removal of the tumor under endoscopic view. **(I)** Post-operative CT revealed opening (purple arrow) of the posterior IAC and medial wall of the bone matter (green arrow) adjacent to the jugular bulb. FIESTA-C, heavily T2-weighted fast imaging employing steady-state acquisition with cycle phase; T1WI, T1 weighted image.

**Figure 5:**
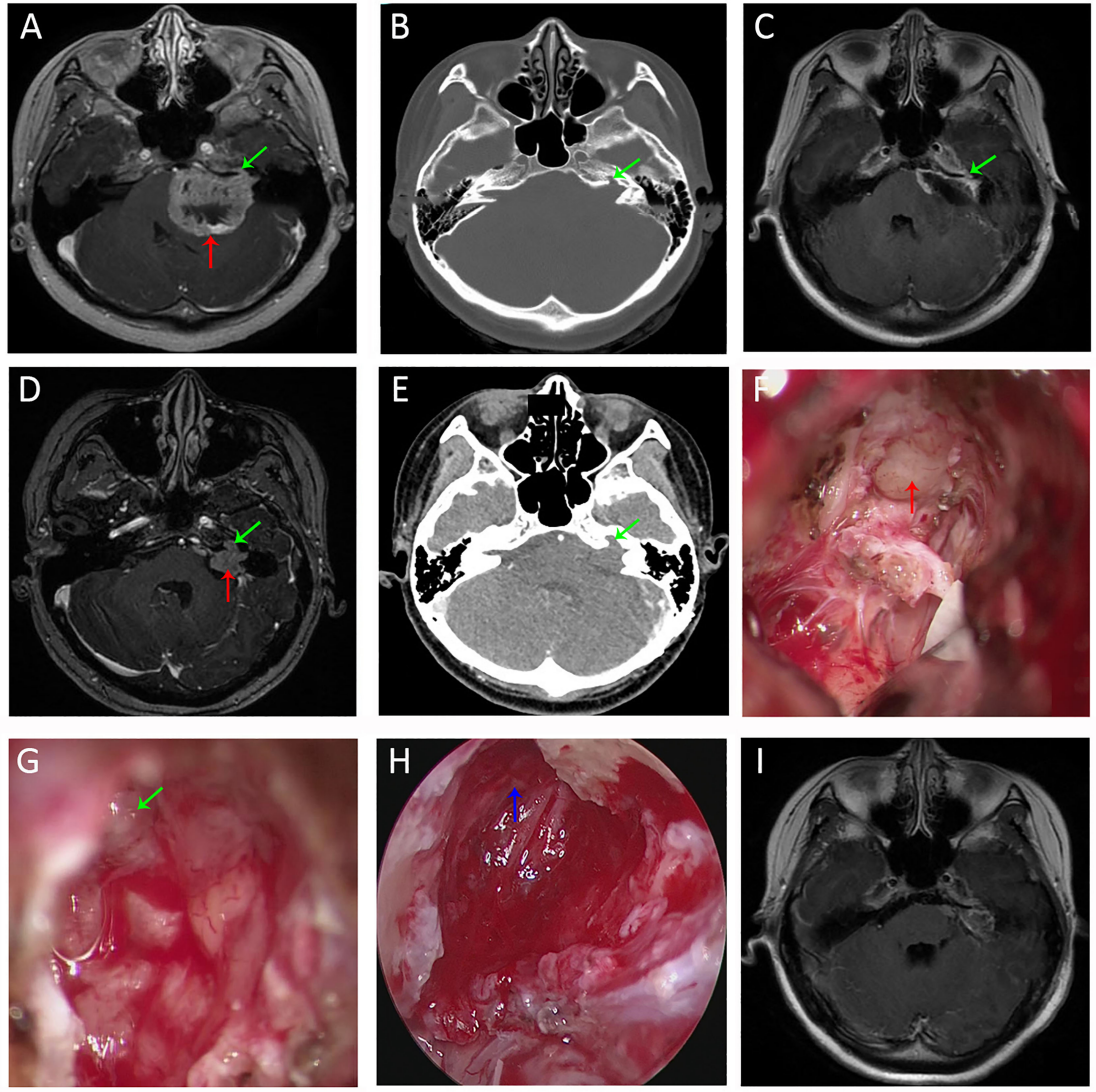
Imaging studies and intra-operative snapshots of a case of vestibular schwannoma with irregularly shaped tumor extending into the IAC. **(A)** The initial pre-operative T1WI with contrast revealed a giant vestibular schwannoma abutting the brainstem (red arrow). The intracanalicular portion of the tumor was irregularly shaped as indicated by the green arrow. **(B)** The bone proper of the fundus of IAC was eroded (green arrow). **(C)** TIWI with contrast after microscopic resection revealed tumor remnants (green arrow) within the bony corridors of the eroded IAC fundus. **(D)** Pre-operative T1WI of the second operation (3 years after the initial surgery) revealed recurrent tumors within IAC. The intracanalicular portion was irregularly shaped, and the extracanalicular portion had extension into CPA. **(E)** Pre-operative CT of the second operation revealed further destructions of the IAC fundus compared with the first operation **(B)**. **(F)** Intra-operative microscopic view of the second operation showed tumor extension to CPA. **(G)** There were still tumor remnants (the irregularly shaped intracanalicular portion, as annotated by green arrow) despite maximal resection under microscopic view. **(H)** Full removal of the tumor remnants (blue arrow) within the bony corridors of IAC. **(I)** Post-operative T1WI of the second operation indicated total removal of the intracanalicular portion of the tumor. Strict adhesions anterior to the brainstem prevented us from further resection. T1WI, T1 weighted image. CPA, cerebellopontine angle; IAC, internal acoustic canal; T1WI, T1 weighted image.

Of the 61 cases, 60 cases (98.36%) achieved gross total resection and 1 case (1.64%) underwent sub-total resection (**Table 1**). Intracanalicular remnants were found and completely removed in 4 cases after endoscopic inspection. The proportion of intracanalicular remnants in Grade B (4/23, 17%) was significantly higher (Fisher’s exact; p = 0.02) than that of Grade A (0/38). HJB was identified in one of the four remnant cases (**Figure 4**). Another patient presented with recurrent tumor invading the adjacent bone proper. The fundus of IAC was eroded, which conferred an irregular shape on the tumor. The narrow corridors were navigated using neuroendoscope to facilitate resection (**Figure 5**). CN VII and VIII were anatomically preserved in all patients. Regarding post-operative HB scale, 50 cases (81.97%) were Grade 1, 5 cases (8.20%) Grade 2, 4 cases (6.56%) Grade 3, and 2 cases (3.28%) Grade 4 (**Table 1**). The results of hearing outcomes are beyond the scope of this article.

## Discussion

Gross total resection has been associated with improved quality of life and potentially long-term control of vestibular schwannomas (3, 5, 6). Most vestibular schwannomas have intracanalicular portions. One of the most common causes of vestibular schwannoma recurrence is remnants at the fundus of IAC (6, 7). To achieve maximal resection for tumors with intracanalicular extension, care must be taken to prevent concomitant injuries to the posterior SCC, the endolymphatic duct, and a potential HJB (8, 9). Injuries to the former two structures may cause hearing loss, while injuries to the later may lead to hemorrhage and air embolisms (9). Retrosigmoid approach, a workhorse in surgical management of CPA lesions, is a versatile option in terms of preserving functions in most tumor sizes (10–13). However, the preservation rate of CN VII functions using retrosigmoid approach vary from 80 to 92% in tumor sizes of 1–2 cm to 50–76% in those > 2cm (14). Many factors, namely, surgical skills, tumor sizes, tumor extensions (intracanalicular and/or extracanalicular), cystic characteristics, and the topography of IAC may all or in part contribute to worse CN VII outcomes (15–23). Due to a relatively smaller sample size, we did not find any pre-operative CN VII dysfunctions in our sample. This would in effect diminish the impact of pre-operative HB scale on post-operative HB scale.

To reach the intracanalicular portion, drilling the posterior wall of IAC is one of the key steps in retrosigmoid approach (8, 24). The surgical nuance here is to ensure maximal exposure of the posterior wall of IAC with protection of neighboring structures. In a healthy individual, the shape of porus acusticus is oval or elliptical (25). The average length of IAC ranges from 5.5 to 12.3 mm, mean diameter 4 mm, according to the present literature (26, 27). To date, the standard of optimal drilling range has not been established. At least three parameters are required to define a drilling range: the length of lateral drilling, the length of the drilled posterior wall of IAC, and the drilling angle. The length of lateral drilling was proposed by some experts to be 10 ± 2 mm (28). The length of drilled posterior wall of IAC ranged from 5 to 10 mm, according to the present literature (28–30). Using frameless navigation on 10 cadaveric heads, Pillai et al. concluded the angle of drilling as 43.3 ± 6.0˚ (24). In our experience, we made a “flexible rule”, in which the length of lateral drilling was approximately 12–14 mm, drilling angle 43˚ and the length of drilled posterior wall of IAC 8 mm (**Figure 2**). After about 8 mm of the posterior wall of IAC was drilled, there were still blind spots at the lateral end of IAC under microscopic view. If tumor remnants were located there, the risk of nerve injuries under “blind” manipulations would increase. Therefore, the application of an endoscope should theoretically minimize the risks of nerve injuries caused by “blind dissection”.

Many reports of endoscopic usage in vestibular schwannomas and/or other CPA lesions were seen in literature (24, 31–33). The purely endoscopic approach requires fewer brain retractions, and the advantage of increased visualization at IAC by navigating through narrow corridors would minimize the risks of complications (34). However, the usefulness of purely endoscopic approach for vestibular schwannomas is still in debate (34–36). Having an assistant to hold the endoscope with both hands may overcome the single-handed technique by the operator, but the dynamic movement of the endoscope requires constant searching for a pivoting point for balance. The bulky manipulation by two hands and the time-consuming nature of endoscopic surgery may compromise visual stabilization. A pneumatic endoscope holder is surely more versatile but less flexible than freehand manipulation. In addition, navigating solely by endoscope is prone to disorientation and concomitant injuries to the vital and complex neurovasculature of CPA (37). Therefore, microscopic resection is still the mainstay of treating vestibular schwannomas.

**Figure 6:**
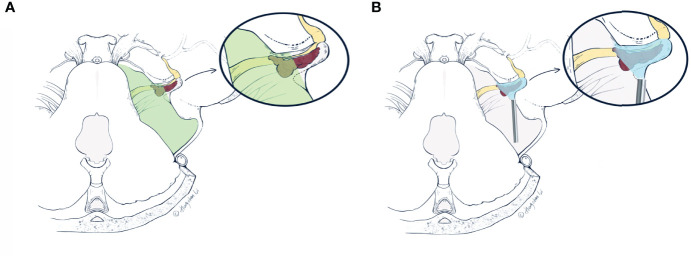
The artist’s (Hongchan Li) illustrative comparisons of differences in visualizations of the retrosigmoid approach under the microscope **(A)** and neuroendoscope **(B)**, respectively. The vestibular schwannoma (red) is first debulked at the porus acusticus, and the posterior IAC is drilled based on pre-operative planning. Drilling range of the posterior wall of IAC is delineated by dashed lines (in **A**, **B**) as a triangle area from an axial view. **(A)** The microscopic view (green area) is limited by the bony anatomy of the retrosigmoid approach, which prevents further resection of the intracanalicular portion. These tumor remnants within the IAC tend to recur and erode adjacent structures. **(B)** The endoscopic viewing area (blue area) includes both extra- and intracanalicular portions. With a 0- or 30-degree endoscope, the area difficult for microscopic view within IAC can be more readily visualized, which facilitates gross total resection of the tumor. IAC, internal acoustic canal (Used with permission from Hongchan Li).

Using a neuroendoscope after microscopic phase is the optimal tactic to circumvent these problems. The surgery should always start with microscopic dissection, which is familiar and less time-consuming for many neurosurgeons. For tumors with less extension into IAC, microscopic dissection would be adequate. While in tumors with ≥10 mm extension into IAC, we would opt for an extra endoscopic phase. The optimal visualization (**Figure 6**) brought by the neuroendoscope would identify potential tumor remnants and facilitate gross total resection using angled instruments (35, 36). If a routinely performed 8 mm of drilling is not enough for more laterally located tumors, an extra 2 mm could be drilled under endoscopic visualization to manage more laterally located tumor remnants.

In practice, neuroendoscopy involves a significant learning curve that demands extensive training. During the surgery, the assistant needs to be familiar with not only the procedure, but also the preferences of the operating surgeon. It is also important for the assistant to irrigate the neuroendoscope to avoid debris accumulation on the tip and thermal injuries to nerves. Above all, in the hands of an experienced neurosurgical team, endoscope-assisted microsurgery of vestibular schwannomas should be considered as a safe and feasible treatment modality, especially for tumors with lateral extension over 10 mm into IAC. Due to a limited sample size, these findings are only preliminary. Further studies are needed to validate the cutoff line of the switching-to-endoscope tactic during microsurgical resection of vestibular schwannomas *via* the retrosigmoid approach.

## Conclusions

In Grade B vestibular schwannomas, after maximal microsurgical removal, endoscopic evaluation of the intracanalicular portion revealed residual tumors in 17% of the patients. Hence endoscopic evaluation of the potential intracanalicular remnants for tumor extending over 10 mm within IAC is recommended.

## Data Availability Statement

The raw data supporting the conclusions of this article will be made available by the authors, without undue reservation.

## Ethics Statement

The studies involving human participants were reviewed and approved by the Institutional Review Board of Shanghai General Hospital. Written informed consent to participate in this study was provided by the participants’ legal guardian/next of kin.

## Author Contributions

YB, YN, DG, and ML conceived of and directed the study. QinZ, QiaZ, and JT provided clinical information and analysis the data. JL and FS participated in data acquisition. HL was responsible for data acquisition and figure productions. JY and YL reviewed the manuscript. All authors contributed to the article and approved the submitted version.

## Funding

This work was supported by the National Natural Science Foundation of China (No. 81902529), the Health Technology Project of Pudong New District Health Committee (PW2020B-2), the Scientific Research Funding Project of Shanghai University of Medicine & Health Sciences Affiliated Zhoupu Hospital (ZPXM-2019A-01), and the Cross Research Fund of Medicine and Engineering of Shanghai Jiao Tong University (Nos. YG2017QN29 and YG2021QN90).

## Conflict of Interest

The authors declare that the research was conducted in the absence of any commercial or financial relationships that could be construed as a potential conflict of interest.

## Publisher’s Note

All claims expressed in this article are solely those of the authors and do not necessarily represent those of their affiliated organizations, or those of the publisher, the editors and the reviewers. Any product that may be evaluated in this article, or claim that may be made by its manufacturer, is not guaranteed or endorsed by the publisher.
