# Corrigendum: Endoscope-Assisted Retrosigmoid Approach for Vestibular Schwannomas with Intracanalicular Extensions: Facial Nerve Outcomes

**DOI:** 10.3389/fonc.2022.963829

**Published:** 2022-06-22

**Authors:** Yunke Bi, Yunjia Ni, Dandan Gao, Qingwei Zhu, Qiangyi Zhou, Junjia Tang, Juan Liu, Fei Shi, Hongchan Li, Jian Yin, Yaohua Liu, Meiqing Lou

**Affiliations:** ^1^ Department of Neurosurgery, Shanghai General Hospital, Shanghai Jiao Tong University School of Medicine, Shanghai, China; ^2^ Department of Neurosurgery, Shanghai General Hospital of Nanjing Medical University, Shanghai, China; ^3^ Department of Hematology-oncology, Zhoupu Hospital, Shanghai University of Medicine and Health Sciences, Shanghai, China

**Keywords:** vestibular schwannomas, neuroendoscope, retrosigmoid approach, internal acoustic canal, facial nerve function

In the published article, there was an error in affiliation 2. Instead of “Department of Hematology-oncology, Zhoupu Hospital, Shanghai University of Medicine and Health Sciences, Shanghai, China”, it should be “Department of Neurosurgery, Shanghai General Hospital of Nanjing Medical University, Shanghai, China”. In addition, the affiliation number of “Department of Hematology-oncology, Zhoupu Hospital, Shanghai University of Medicine and Health Sciences, Shanghai, China” should be 3 instead of 2.

The authors apologize for these errors and state that these do not change the scientific conclusions of the article in any way. The original article has been updated.

## Publisher’s Note

All claims expressed in this article are solely those of the authors and do not necessarily represent those of their affiliated organizations, or those of the publisher, the editors and the reviewers. Any product that may be evaluated in this article, or claim that may be made by its manufacturer, is not guaranteed or endorsed by the publisher.

